# Supplementary material for: Symbolic regression of generative network models
Source: Sci Rep. 2014 Sep 5;4:6284. doi: 10.1038/srep06284 (PMC4155339; doi:10.1038/srep06284)
Supplement: Supplementary Information [file srep06284-s1.pdf]

Supplementary Information for “Symbolic regression of generative network models”

Telmo Menezes, Camille Roth

Contents

1 Interpretation **2**

2 Benchmarks **8**

3 Comparison with previous approaches **11**

4 Algorithms and detailed parameters **13**

5 Numeric results **20**

1 Interpretation

1.1 C.elegans

$$w(i,j) = \log(d_D)^{d_D - 7} - \min(j + 0.52, 0.77)$$

The C.elegans generator uses two variables: d_D and j. Given the term where j appears, $min(j + 0.52, 0.77)$, a distinction is only made between $j = 0$ and $j > 0$. For the first case, the term takes the value 0.52, for the second the value 0.77. The first term uses the d_D variable, which takes integer values in $\{0, ...5\}$. Only values equal to or greater than 2 are relevant, given that $d_D = 0$ configures a self-link, while $d_D = 1$ indicates an already existing arc. Both these situations are prohibited in the configuration we use.

As negative values are considered null weights, we can reduce the generator to 3 possible situations, shown in table 1.

Here we can see that the rule is indeed very simple: the generator promotes the creation of $A \to B$ arcs where an $A \to C \to B$ arc already exists ($d_D = 2$). For the special case of an arc where the target has the sequential identifier 0, $d_D = 3$ is also allowed, albeit with a much lower probability. For $d_D = 2$, connection to this special node are also slightly preferred.

Sequential identifiers are abstractions meant to capture some *a priori* heterogeneity in the vertex set, so the meaning of the special node 0 can only be speculated on. It might represent a certain spatial constraint for a neuron or a different behaviour of the neuron through some mechanism. Such interpretations are beyond the scope of the work and better left to researchers

d_D	$j = 0$	$j > 0$
2	5.73	5.48
3	0.17	0
4	0	0
5	0	0

Table 1: Weights table for C.elegans generator.

with the adequate domain knowledge.

1.2 Political Blogs

$$w(i, j) = \exp(4 - 2d)$$

The political blogs generator takes a single variable: d. Unlike with d_D, the case where $d = 1$ can happen, as this distance may be caused by an arc with the opposite direction of the one being proposed. This generator behaviour can be simply described, as shown in figure 1.

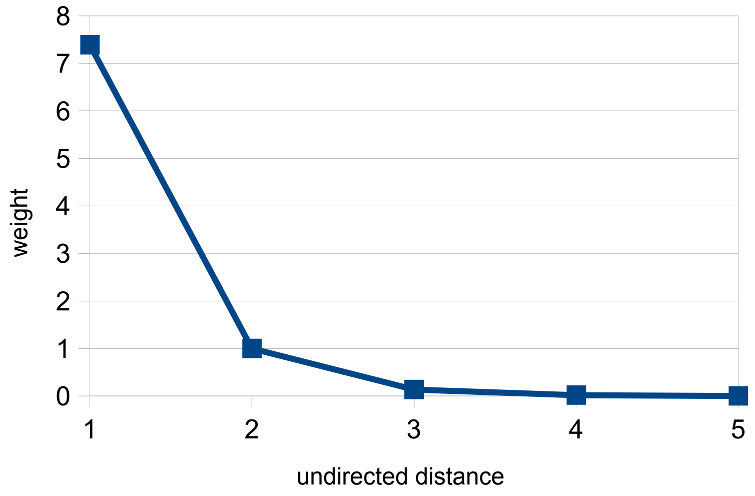

Figure 1: Weight of political blogs generator as a function of the undirected distance.

Reciprocity is strongly encouraged, given the high weight given to arcs with $d = 1$. In fact, this situation necessarily corresponds to a reciprocal arc (linking to a blog that linked to you), because arcs that already exist are not allowed in the sample. Then, weight decreases as undirected distance increases, but never to 0. This distribution of weights according to undirected distances is sufficient to generate the two communities in the network — links between blogs that have a low undirected distance are more probable, but links to more distant blogs are also possible. This leads to the low density of links between the two communities. Surprisingly, it

is possible to propose a plausible generator that leads to the two communities without resorting to *a priori* diversity (e.g Democrats vs. Republicans), that could be expressed with sequential identifiers or the affinity function. It can be speculated that this matches the way in which political ideology propagates in society.

1.3 Software Collaborations

$$\mathrm{w}(i,j) = \psi\left(\frac{k'(i)}{\mathrm{d}}, k'(j), 0.7k(j)\right) \cdot \min\left(k(j), 9\right)$$

The software collaborations network represents software authors from the CPAN Perl language repository. An arc means then an author uses a software module from another author.

Here we can see that the affinity operator is used. The first parameter of the operator represents the number of groups that the vertex set is divided into, so the higher this value, the less likely affinity between to arbitrary vertices becomes. The number of groups is rounded to the closest integer.

As the number of groups is determined by $\frac{k'(i)}{\mathrm{d}}$, an increase in the out-degree of the origin makes affinity less likely and an increase in undirected distance makes it more likely. The expression can be divided into two branches:

- *affinity*: $k'(j) \cdot \min\left(k(j), 9\right)$
- *no affinity*: $0.7k(j) \cdot \min\left(k(j), 9\right)$

Both branches represent a form of preferential attachment. The first one uses a combination of the in- and out-degree of the target, while the second only considers the in-degree.

A plausible interpretation is that the generator proposes two modes of discovery: by proximity in the network or by problem domain affinity. Affinity is more likely when the origin is still an outsider to the environment (low number of dependencies on other authors' work) or when the topological distance is high – maybe an existing author expanding to a new problem

domain. When module discovery is by proximity, the only thing that is taken into account is the number of authors that already depend on the work of the target — its popularity for a certain problem domain. When it is by affinity, the number of authors that it depends on also factors in. This could represent a measure of integrative power — how much the work of a certain author encapsulates existing solutions in a higher abstraction, providing a more useful entry point for a newcomer to its domain.

1.4 Power Grid

$$\mathrm{w}(i,j) = \psi\left(\mathrm{d}^{\mathrm{d}\cdot\begin{cases} i-1, & \text{if } \mathrm{k}(j)=0 \\ \mathrm{k}(i), & \text{otherwise} \end{cases}}, 1, 0 \right)$$

Here we start addressing the undirected networks. The power grid generator also uses the affinity generator, but in a more binary fashion: the weight is always 1 if there is affinity and null if there is not. All the complexity of the generator is captured by the decision on the number of affinity groups. The following things can be noted about the expression that determines the number of groups:

- Overall, the probability of affinity decreases exponentially with distance.
- The node with the sequential identifier 1 overrides the previous rule if the target has no connections, by making the exponent null. This configures a central hub for newcomers to connect to.
- An origin with no connections overrides the previous rule if the target is already connected, also by making the exponent null.

This configures a branching growth behaviour, where new vertices either connect to a central hub or some already connected vertex, but connections between already connected or distant

vertices are discouraged. The tree-like structure that originates from this matches our intuitions about the growth of a power grid. The affinity operator, by implicitly using sequence identifiers, might abstract at a high level the underlying spatial constraints.

1.5 Facebook

$$\mathrm{w}(i,j) = \psi\,(3, i \cdot \mathrm{k}(i), \mathrm{k}(i))$$

The facebook generator combines preferential attachment with affinity in a simple fashion. The constant value for the number of groups suggests pre-existing social circles.

- *affinity*: $i \cdot \mathrm{k}(i)$
- *no affinity*: $\mathrm{k}(i)$

The role of the factor i in case of affinity appears to be two-fold: increase the propensity for connections inside pre-existing social circles (for the most cases $i > 1$) and capture an external preference for certain connections within such groups over others. This matches the intuition that popularity in the real world is expressed in the facebook social graph.

Connections between groups follow a pure preferential attachment regime, matching the intuition that more popular individuals are more likely to act as bridges between communities.

1.6 Proteins

$$\begin{cases} \log(i), & \text{if } \mathrm{k}(j) = \begin{cases} 0, & \text{if } \mathrm{k}(i) < 4 \\ \mathrm{k}(i), & \text{otherwise} \end{cases} \\ -1, & \text{otherwise} \end{cases}$$

The protein interactions generator only assigns a non-null weight in two cases: if the target is not yet connected and the origin has a low degree ($k(i) < 4$), or if the degree of the origin and target are the same. Non-null weights are always $\log(i)$, indicating an *a priori* logarithmically distributed propensity for interaction.

This generative process seems to indicate a strong preference for assortativity on degree, establishing edges between proteins that interact with a similar number of other proteins.

1.7 Word Adjacencies

$$\mathrm{w}(i,j) = (k(i) - \mathrm{d})$$

This network represents the adjacencies of common adjectives and nouns in the novel "David Copperfield" by Charles Dickens.

The generator configures a simple combination of preferential attachment with a preference for connecting to close vertices. An increase in the degree of a node also increases its reach, as the distance term can be higher with making the weight negative, and thus null.

2 Benchmarks

In this section we present the detailed results obtained from performing evolutionary search on networks produced by two known generators: preferential attachment (PA) and random (ER). For each type of generator we perform a total of 150 runs, with 30 runs for each of 5 different network sizes. We analyse both the correctness of the results and the computational run time. We also present the run times and number of generations for the real-world networks that were used in this work.

For the artificial networks we found a correct results rate of 97.3% for preferential attachment and 94% for random.

In correct results, we distinguish between *perfect* results and *bloated* results. A bloated generator is one with redundant operations, but equivalent to the perfect solution. To illustrate, the perfect solution for preferential attachment is:

$$w_{\text{PA}}(i, j) = \text{k}(j)$$

An example of a bloated solution is:

$$w_{\text{PA}}(i, j) = \text{k}(j) + 0$$

The rate of perfect results found was of 92.7% for preferential attachment and 76.7% for random. Figure 2 shows the detailed results according to network size.

To evaluate the impact of network size on the run time of the search algorithm, we timed each one of the evolutionary runs using the UNIX command *time*. Figure 3 shows the outcome of this benchmark. We present a box plot for the *user* measurement produced by the *time* command (*user* is the amount of time spent in user-mode, so it disregards time wasted by other unrelated processes).

Figure 4 shows the run times for the several real-world networks as well as the number of generations until the stopping condition is achieved.

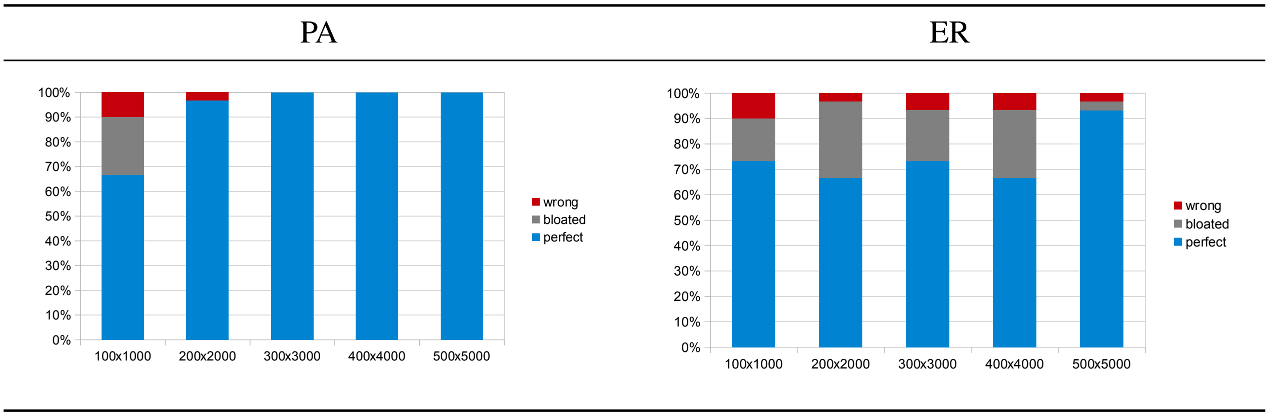

Figure 2: Results of evolutionary runs over networks of different sizes produced respectively by a preferential attachment (PA) generator (*left*) and by a random (ER) generator (*right*). Network sizes represented in vertices x arcs.

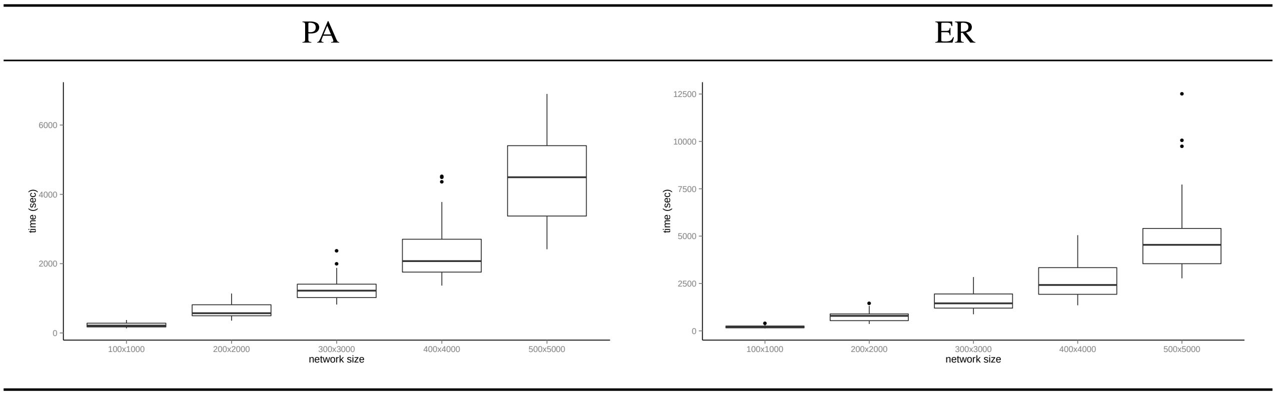

Figure 3: Impact of network size on run time – preferential attachment (*left*) and random (*right*).

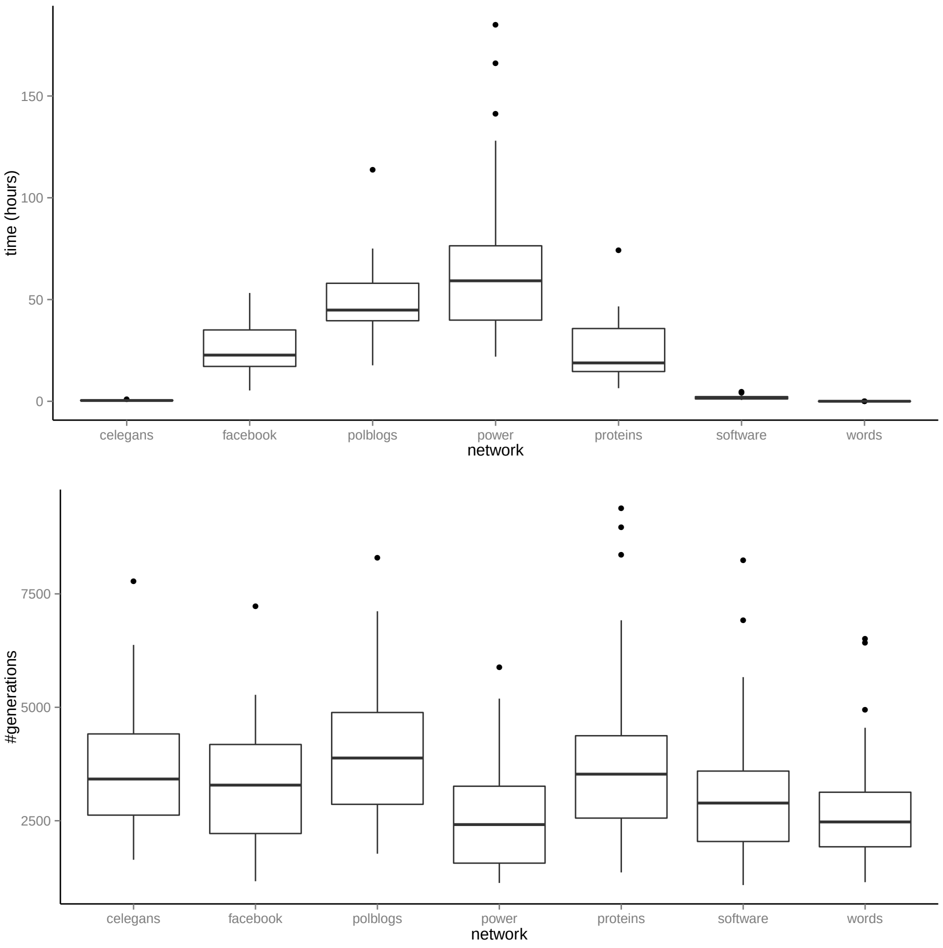

Figure 4: Run times and generations for each real-world network.

3 Comparison with previous approaches

Bailey et al. (*4, 5*) propose a genetic programming approach to finding generators for undirected networks. Their approach focuses solely on finding generators for synthetic networks (random, preferential attachment and small-world). For random networks, for example, it finds relatively complex solutions that vary with the target network size. We present results from using our approach in both synthetic networks and a variety of real-world cases. We show that, for the synthetic cases, the simplest (exact) solution is found the majority of the times and the same optimal solution is found independently of network size. In addition, we show that generators converge on similar solutions for a real-world case. The fitness function proposed in that method relies on empirical assumptions about the relative weight of the several metrics used in the fitness function, while the fitness function we propose makes no such assumptions.

Methodologically, our approach is also significantly different in that the above-mentioned work defines generators with three steps – initialisation, growth and finalisation – and rely on several types of actions (e.g creation of a ring of nodes, triangles of nodes, edge removal and edge rewiring). The generators are influenced by both local and network-level information. Examples of the latter include average and maximum degree, total vertex count and total edge count. In contrast, we propose a simpler generator system that relies on a single step and a single type of action: edge/arc creation, and that uses only local information. We propose that our approach leads to generators that are easier to understand, with no complicated interactions between generation steps or types of actions, and that can be directly mapped to conventional mathematical expressions. Furthermore, we propose that initialisation with structures, edge removal and rewiring lead to a larger space of possible solutions, from which is not possible to choose solely on the basis of a static snapshot of a network. By employing only local information, our approach finds solutions that more plausibly match decentralised dynamics, that are more likely to be found in biological and social systems, and which are precisely the hardest to

model.

For these reasons, we claim that our work is an important step forward in the explanatory power of the generators, the applicability to complex network research and the extensibility of the methodology to specific domains – by easily adding other metrics to the fitness function or local information to the generators.

4 Algorithms and detailed parameters

In this section we present the main algorithms used in this work in pseudo-code. The complete code is available at `https://github.com/telmomenezes/synthetic`.

Topological distance computation. As said in the main text, the explicit computation of pairwise distances is computationally extremely expensive. Instead, distances are determined by random walkers that are embedded in the generative process. There is a random walker for each node in the network. Each walker starts on its node and takes one step per generator cycle (one generator cycle corresponds to the creation of a new edge in the network). Walkers have a maximum length and go back to their nodes when this length is reached. We use 6 in our experiments, as distance does not appear to play a significant role above this value (*6*). For every node visited by a walker, the current length of the walk is compared to the current distance between origin and current node. The walk length becomes the new distance if it is lower. When dealing with directed networks we have both directed and undirected distances. In this case we have both types of walkers. Undirected walkers are allowed to step through arcs in any direction. This heuristic-based distance definition is admittedly distinct from the exact topological distance, yet it might also actually be more realistic in that it mimics a myopic exploration behaviour which is likely to be playing a significant role in decentralised, self-organised network construction processes.

Distribution dissimilarity computation. For degree and PageRank centralities we apply the *Earth mover's distance (EMD)* and associated algorithms (*1*). This algorithm is based on an analogy between distributions and piles of dirt. The distance is the minimum cost of turning one pile into the other, where the cost is the amount of moved dirt multiplied by the distance by which it is moved. It makes sense to use EMD where moving entities between bins in the distributions tends not to have an impact on the rest of the distribution (e.g. switching the origin

of one arc from one node to another increments the value in one bin by one and decrements the value on another bin by one). This is not so straightforward for centrality distributions, but we still found it to work well in this case.

For the more sophisticated distance distributions and triadic profiles (i.e., triadic pattern distributions), we rely on a simpler ratio-based dissimilarity metrics as there are more global side-effects to moving entities. The dissimilarity metrics is essentially a sum over all distribution bins $i \in 1,..,B$ of the difference between counts of the synthetic bin b_i and the target bin b'_i, relative to the counts of the synthetic bin (when non-zero):

$$d(b, b') = \sum_{i=1}^{B} \frac{|\#b_i - \#b'_i|}{n_0(\#b_i)}$$

where n_0 is a helper function intended at preventing divisions by zero: $n_0(x) = x$ if $x \neq 0$, and 1 otherwise.

Program tree initialisation and mutation. As mentioned in the main text, the evolutionary search algorithm is initialised with a randomly created generator. The random initialisation algorithm we use employs a number of common techniques from the genetic programming literature. The goal is to attain variety, but it is likely that the specific details and parameterisation have little impact on the outcomes of the search algorithm.

For each tree that is generated, a minimum depth md is randomly and uniformly selected between md_l and $md_h \in \mathbb{N}$. One of two generative processes is selected with equal probability: *fixed depth* or *grow*. In *fixed depth*, function nodes are randomly selected for tree nodes below the minimum depth and terminal nodes are randomly selected for tree nodes at the minimum depth. Terminal nodes are either constants or variables. In *grow*, function nodes are randomly selected for tree nodes below the minimum depth. After that, there is a pre-defined probability p_{term} that a terminal node is selected. The former method produces balanced trees while the latter generates more irregularity in terms of branch depths.

Variable	Name	Directed/Undirected
i, j	Sequential vertex identifiers	both
k	In-degree / degree	both
k'	Out-degree	directed
d	Simple distance	both
$\mathrm{d_D}$	Direct distance	directed
$\mathrm{d_R}$	Reverse distance	directed

Table 2: List of generator inputs.

Terminal nodes are randomly selected to be either a variable or a constant with equal probability. In the former case, a variable is selected from the variable set with equal probabilities. In the latter, there is a 0.1 probability that the constant 0 is elected, a 0.4 probability that an integer from a uniform distribution in $\{0, ..., 9\}$ is selected and, finally, a 0.5 probability that a real value from a uniform distribution in $[0, 1]$ is selected. This is meant to encourage the appearance of constant building blocks that are intuitively expected to be useful.

The mutation process consists in the random uniform selection of a node in the program tree to be mutated, the removal of this node and the sub-tree that may exist underneath it, and its replacement with a new, randomly generated sub-tree. The new sub-tree is extracted from another program that was generated for this purpose using the same strategy previously described. Both in the original program and in the randomly generated auxiliary program, the nodes that indicate the root of the sub-trees to use are randomly selected from the set of all nodes in the tree with equal probability for each node. This mutation strategy is usually referred to as *headless chicken mutation* in the genetic programming literature (*2, 3*).

The set of variables that might be present in a generator is enumerated in table 2. The parameter values used throughout all the experiments presented in this work, including md_l, md_h and p_{term}, are listed in table 3.

Parameter	Value
Stable generations stopping condition	1000
Anti-bloat tolerance	0.1
Sample ratio	0.0006
Number of bins for continuous distributions	100
md_l	2
md_h	5
p_{term}	0.4

Table 3: List of parameters.

Network generators and comparison algorithms. We now describe the various algorithms in detail. Algorithm 1 is the main evolutionary search loop. It corresponds to the `run()` method in the `synthetic.Evo` Java class.

The algorithm references several functions. Some of them (`randomGenerator()`, `clone()`, `mutate()`) manipulate program trees in conventional ways that are thoroughly described in the genetic programming literature.

`isBetterThan()` is used to tolerate a small decrease in fitness in exchange for a shorter generator program. This function is presented as algorithm 2 and corresponds to the `synthetic.generators.Generator.isBetterThan` method in the source code.

`computeFitness()` is presented as algorithm 3. This function simply generates a synthetic network and computes its dissimilarity to the target network, using the metrics and approach described in the main text.

`generateNet()` is presented as algorithm 4. This describes how a generator is used to produce a synthetic network. Both these function are implemented in `synthetic.generators.Generator`.

More specifically, algorithm 5 describes the process behind each arc creation step.

`setGeneratorVariables()` is a function that assigns the appropriate local values to the variables in the generator program.

```
Data: a network (net), stable generations stop condition (maxGens), anti-bolat
      tolerance (tol)
Result: a generator program
bestFitGenerator ⟵ randomGenerator();
bestGenerator ⟵ bestFitGenerator;
bestFitness ⟵ computeFitness(bestFitGenerator, net);
stableGens ⟵ 0;
while stableGens < maxGens do
    stableGens ⟵ stableGens + 1;
    if random value ∈ [0, 1] < 0.5 then
        newGenerator ⟵ clone(bestFitGenerator);
    else
        newGenerator ⟵ clone(bestGenerator);
    newGenerator ⟵ mutate(newGenerator);
    newFitness ⟵ computeFitness(newGenerator, net);
    if newFitness < bestFitness then
        bestFitness ⟵ newFitness;
        bestFitGenerator ⟵ newGenerator;
        stableGens ⟵ 0;
    if isBetterThan(newGenerator, bestGenerator, bestFitness, tol) then
        bestGenerator ⟵ generator;
        stableGens ⟵ 0;
return bestGenerator
```

Algorithm 1: Evolutionary search.

```
Data: two generator programs (gen1 and gen2), best fitness so far (bestFitness),
      anti-bloat tolerance (tolerance)
Result: true if gen1 is better than gen2, false otherwise
fit1 ⟵ fitness of gen1;
fit2 ⟵ fitness of gen2;
size1 ⟵ program size of gen1;
size2 ⟵ program size of gen2;
if |fit2 - bestFitnessMax| < tolerance then
    return false
if |fit1 - bestFitnessMax| < tolerance then
    return true
return size2 < size1
```

Algorithm 2: Which generator is the best? (*isBetterThan(gen1, gen2, bestFitness, tol)*)

```
Data: a generator program (gen), target network (net)
Result: a network
verts ⟵ number of vertices of net;
arcs ⟵ number of arcs of net;
dir ⟵ is net directed?;
syntheticNet ⟵ generateNet(gen, verts, arcs, dir);
d ⟵ compute distance between net and syntheticNet;
return d
```

Algorithm 3: Compute fitness. (*computeFitness(gen, net)*)

```
Data: a generator program (gen), number of vertices (verts), number os arcs (arcs),
      directed network? (dir)
Result: a network
net ⟵ createEmptyNetwork(dir);
for i = 1 to verts do
    add a vertex to net
for i = 1 to arcs do
    newArc ⟵ genStep(gen, net);
    addArc(net, newArc);
    random walks step;
```

Algorithm 4: Generate network. (*generateNet(gen, verts, arcs, dir)*)

```
Data: a generator program (gen), a network (net)
Result: a network
totalWeight ⟵ 0;
sampleArcs ⟵ array of size trials;
sampleWeights ⟵ array of size trials;
for i = 1 to trials do
    repeat
        arc ⟵ randomArc(net);
    until arcExists(net, arc);
    sampleArcs[i] ⟵ arc;
    setGeneratorVariables(gen, net, arc);
    weight ⟵ eval(prog);
    if weight < 0 then
        weight ⟵ 0;
    sampleWeights[i] ⟵ weight;
    totalWeight ⟵ totalWeight + weight;
if totalWeight = 0 then
    for i = 1 to trials do
        sampleWeights[i] ⟵ 1;
        totalWeight ⟵ totalWeight + 1;
targWeight ⟵ rand() * totalWeight;
i ⟵ 0;
totalWeight ⟵ sampleWeights[i];
while targWeight > totalWeight do
    i ⟵ i + 1;
    totalWeight ⟵ totalWeight + sampleWeights[i];
return sampleArcs[i];
```

Algorithm 5: Generator step. (*genStep(gen, net)*)

5 Numeric results

In this section we provide detailed results for the experiments run in the context of this work.

Four tables are provided with the mean values for the several dissimilarity metrics we use to derive fitness values: k and k' correspond to metrics relative to degree centrality distributions, PR_d and PR_u to directed and undirected PageRank centralities, τ to triadic profiles, $\mathrm{d_D}$ and d to the respective topological distances ($\mathrm{d_R}$ is equivalent to $\mathrm{d_D}$ at the distributional level). Tables 4 and 5 present values relative to random networks with the same number of vertices and arcs as the target network. Tables 6 and 7 present the absolute values. All tables also include program sizes (p_S).

**Network**	**Model**				**Distances**						
	$prog$	f_{max}	f_{mean}	p_s	k_{in}	k_{out}	PR_d	PR_u	τ	d_d	d_u
**C. Elegans**	*p00*	0.53	0.32	25	0.51	0.48	0.39	0.24	0.25	0.46	0.20
	p01	0.56	0.42	14	0.82	0.51	0.29	0.41	0.29	0.52	0.51
$m = 297$	*p02*	0.64	0.50	10	1.05	0.49	0.54	0.44	0.39	0.64	0.50
$n = 2345$	*p03*	0.64	0.50	4	1.05	0.48	0.55	0.45	0.38	0.64	0.51
	p04	0.51	0.38	13	1.03	0.35	0.32	0.33	0.48	0.50	0.22
	p05	0.75	0.57	9	0.77	0.69	0.70	0.42	0.54	0.68	0.58
	p06	0.46	0.28	16	0.44	0.26	0.38	0.22	0.20	0.44	0.28
	p07	0.82	0.54	3	1.30	0.39	0.79	0.24	0.25	0.81	0.69
	p08	0.62	0.49	10	1.04	0.47	0.54	0.45	0.37	0.62	0.51
	p09	0.73	0.54	14	1.50	0.58	0.60	0.18	0.41	0.72	0.58
	p10	0.64	0.41	8	1.11	0.61	0.36	0.35	0.32	0.60	0.10
	p11	0.70	0.50	5	1.06	0.46	0.50	0.34	0.55	0.70	0.47
	p12	0.64	0.50	4	1.05	0.47	0.55	0.45	0.38	0.64	0.52
	p13	0.87	0.55	5	1.27	0.43	0.68	0.25	0.31	0.87	0.71
	p14	0.59	0.46	14	0.82	0.46	0.49	0.32	0.57	0.56	0.46
	p15	0.82	0.50	9	0.77	0.53	0.31	0.59	0.42	0.82	0.45
	p16	0.61	0.41	45	0.87	0.43	0.31	0.32	0.42	0.61	0.38
	p17	0.59	0.47	23	1.16	0.51	0.55	0.27	0.40	0.57	0.48
	p18	0.81	0.54	5	1.30	0.39	0.74	0.28	0.30	0.81	0.66
	p19	0.61	0.47	21	0.98	0.56	0.54	0.38	0.36	0.51	0.52
	p20	0.53	0.42	11	1.01	0.47	0.50	0.31	0.36	0.42	0.44
	p21	0.68	0.46	11	0.75	0.62	0.52	0.28	0.38	0.65	0.46
	p22	0.83	0.53	23	0.66	0.50	0.52	0.56	0.28	0.83	0.72
	p23	0.49	0.32	8	0.59	0.47	0.37	0.32	0.36	0.38	0.10
	p24	0.89	0.51	13	1.48	0.63	0.69	0.15	0.28	0.74	0.41
	p25	0.46	0.28	13	0.26	0.27	0.33	0.20	0.21	0.43	0.43
	p26	0.54	0.43	9	0.87	0.52	0.36	0.43	0.46	0.37	0.50
	**p27**	**0.45**	**0.33**	**11**	**0.52**	**0.44**	**0.31**	**0.33**	**0.34**	**0.31**	**0.33**
	p28	0.63	0.49	10	1.10	0.46	0.57	0.41	0.33	0.62	0.57
	p29	0.79	0.61	7	0.96	0.71	0.68	0.77	0.29	0.72	0.68
**Political Blogs**	*p00*	0.58	0.35	12	2.36	0.37	0.43	0.14	0.18	0.58	0.19
	p01	0.82	0.51	12	3.04	0.35	0.59	0.29	0.60	0.77	0.28
$m = 1490$	*p02*	0.59	0.33	28	2.37	0.30	0.27	0.08	0.31	0.59	0.25
$n = 19022$	*p03*	0.83	0.47	16	2.57	0.28	0.55	0.32	0.35	0.83	0.40
	p04	0.80	0.47	27	2.29	0.35	0.57	0.19	0.42	0.80	0.42
	p05	0.75	0.57	14	2.97	0.56	0.75	0.22	0.54	0.69	0.54

	p06	0.61	0.44	15	2.29	0.58	0.36	0.37	0.34	0.57	0.33
	p07	0.50	0.40	21	1.95	0.42	0.35	0.50	0.34	0.44	0.30
	p08	0.96	0.39	59	1.14	0.34	0.27	0.19	0.29	0.96	0.43
	p09	0.78	0.48	8	2.62	0.47	0.26	0.64	0.46	0.78	0.15
	p10	0.59	0.34	21	1.60	0.27	0.46	0.17	0.19	0.59	0.33
	**p11**	**0.42**	**0.27**	**18**	**1.63**	**0.27**	**0.29**	**0.27**	**0.19**	**0.41**	**0.12**
	p12	0.88	0.71	10	3.43	0.60	0.82	0.51	0.65	0.87	0.71
	p13	0.94	0.39	5	1.29	0.24	0.17	0.28	0.40	0.94	0.40
	p14	0.95	0.53	6	2.74	0.47	0.75	0.25	0.48	0.95	0.17
	p15	0.54	0.39	11	1.86	0.38	0.24	0.43	0.43	0.49	0.32
	p16	0.84	0.32	32	1.85	0.25	0.28	0.09	0.26	0.84	0.07
	p17	0.95	0.43	4	1.60	0.42	0.46	0.36	0.38	0.95	0.08
	p18	0.87	0.53	32	3.03	0.49	0.55	0.43	0.36	0.74	0.48
	p19	0.93	0.59	4	2.70	0.66	0.78	0.38	0.46	0.93	0.31
	p20	0.46	0.27	14	0.87	0.33	0.36	0.15	0.16	0.46	0.24
	p21	0.81	0.60	14	3.21	0.65	0.60	0.49	0.48	0.78	0.45
	p22	0.67	0.31	18	2.15	0.10	0.43	0.12	0.29	0.67	0.09
	p23	0.57	0.32	21	0.81	0.38	0.42	0.32	0.17	0.57	0.19
	p24	0.68	0.45	6	1.01	0.56	0.36	0.67	0.47	0.65	0.21
	p25	0.82	0.39	26	1.94	0.44	0.20	0.22	0.28	0.81	0.37
	p26	0.96	0.59	5	2.87	0.45	0.75	0.22	0.52	0.96	0.55
	p27	1.01	0.92	1	4.17	0.83	1.00	0.79	0.89	1.01	0.97
	p28	0.89	0.36	7	1.55	0.26	0.37	0.26	0.32	0.89	0.09
	p29	0.83	0.61	8	2.49	0.53	0.79	0.17	0.65	0.81	0.72
**Software Collaborations**	*p00*	0.96	0.66	8	2.77	0.44	0.87	0.48	0.57	0.96	0.58
	p01	0.98	0.62	7	1.50	0.58	0.84	0.42	0.51	0.97	0.64
	p02	0.80	0.47	22	1.42	0.46	0.79	0.21	0.46	0.77	0.26
$m = 840$	*p03*	1.01	0.61	7	1.57	0.57	0.84	0.40	0.51	1.00	0.54
$n = 2138$	*p04*	1.09	0.78	11	2.21	0.72	0.92	0.59	0.74	1.05	0.89
	p05	0.92	0.51	9	1.20	0.47	0.87	0.27	0.51	0.73	0.40
	p06	0.90	0.38	7	1.22	0.23	0.89	0.05	0.25	0.83	0.08
	p07	0.97	0.70	3	2.98	0.77	0.70	0.62	0.65	0.97	0.41
	p08	0.95	0.60	11	1.96	0.66	0.94	0.33	0.54	0.92	0.31
	p09	0.91	0.50	11	0.98	0.35	0.75	0.28	0.37	0.91	0.60
	p10	1.04	1.01	1	3.95	1.00	1.02	1.00	1.01	1.03	0.98
	p11	1.01	0.80	12	2.46	0.98	0.83	0.89	0.82	0.99	0.46
	p12	0.80	0.40	6	0.73	0.42	0.78	0.14	0.36	0.71	0.21
	p13	0.93	0.54	5	2.12	0.68	0.55	0.33	0.55	0.93	0.18
	**p14**	**0.69**	**0.33**	**12**	**1.19**	**0.38**	**0.62**	**0.06**	**0.23**	**0.65**	**0.05**
	p15	0.96	0.76	15	2.33	0.71	0.84	0.74	0.73	0.89	0.82
	p16	0.81	0.39	7	1.66	0.40	0.62	0.08	0.29	0.80	0.07
	p17	0.85	0.37	6	1.35	0.32	0.64	0.10	0.25	0.84	0.11
	p18	1.03	1.00	1	3.85	1.00	1.01	1.00	1.00	0.99	1.01
	p19	0.89	0.55	5	1.71	0.46	0.84	0.37	0.57	0.84	0.35
	p20	0.80	0.42	9	0.92	0.56	0.70	0.13	0.38	0.77	0.17
	p21	0.94	0.45	16	1.32	0.20	0.87	0.18	0.44	0.86	0.24
	p22	1.03	1.00	1	3.85	1.00	1.00	1.00	1.00	1.00	1.00
	p23	0.95	0.53	7	0.99	0.46	0.94	0.32	0.49	0.83	0.41
	p24	0.80	0.34	19	1.06	0.31	0.57	0.06	0.28	0.79	0.07
	p25	0.97	0.67	6	2.24	0.64	0.91	0.61	0.73	0.97	0.26
	p26	1.01	0.95	1	3.73	0.96	1.00	0.88	0.95	1.00	0.89
	p27	0.98	0.51	26	0.85	0.52	0.74	0.24	0.36	0.96	0.54
	p28	1.03	1.00	1	3.86	1.00	1.01	1.00	1.00	1.00	0.99
	p29	0.96	0.58	14	1.97	0.39	0.62	0.41	0.40	0.96	0.79

Table 4: Detailed relative results for directed networks. Each line shows the mean values for 30 runs of one generator.

**Network**	**Model**				**Distances**			
	$prog$	f_{max}	f_{mean}	p_s	k	PR	τ	d

**Facebook**	*p00*	0.64	0.56	13	0.62	0.64	0.41	0.59
	p01	0.62	0.48	7	0.50	0.62	0.40	0.41
$m = 747$	*p02*	0.77	0.56	9	0.59	0.76	0.56	0.33
$n = 30025$	*p03*	0.70	0.62	15	0.58	0.54	0.65	0.70
	p04	0.78	0.55	20	0.47	0.38	0.68	0.69
	p05	0.76	0.62	4	0.57	0.74	0.52	0.65
	p06	0.52	0.39	14	0.52	0.31	0.34	0.38
	p07	0.77	0.66	7	0.58	0.56	0.73	0.77
	p08	0.69	0.57	8	0.69	0.58	0.65	0.35
	p09	0.62	0.48	7	0.44	0.56	0.60	0.32
	p10	0.79	0.53	7	0.79	0.61	0.41	0.28
	p11	0.58	0.49	13	0.44	0.44	0.49	0.58
	p12	0.79	0.68	7	0.70	0.54	0.77	0.71
	**p13**	**0.29**	**0.20**	**14**	**0.23**	**0.14**	**0.25**	**0.18**
	p14	0.48	0.35	27	0.30	0.45	0.47	0.18
	p15	0.91	0.52	10	0.51	0.89	0.54	0.16
	p16	0.43	0.30	14	0.31	0.37	0.19	0.31
	p17	0.40	0.30	10	0.33	0.27	0.34	0.26
	p18	0.42	0.35	10	0.39	0.37	0.32	0.33
	p19	0.48	0.36	6	0.45	0.40	0.40	0.20
	p20	0.69	0.40	9	0.44	0.66	0.27	0.23
	p21	0.72	0.52	12	0.56	0.66	0.38	0.48
	p22	0.63	0.43	18	0.22	0.27	0.61	0.61
	p23	1.05	0.55	17	0.46	1.05	0.40	0.31
	p24	0.75	0.63	10	0.54	0.68	0.57	0.73
	p25	0.39	0.28	13	0.33	0.32	0.32	0.14
	p26	0.97	0.72	5	0.54	0.62	0.87	0.85
	p27	0.48	0.37	10	0.38	0.44	0.33	0.32
	p28	0.40	0.28	6	0.34	0.25	0.36	0.15
	p29	0.43	0.32	6	0.38	0.37	0.34	0.19
**Power Grid**	*p00*	1.02	1.00	1	0.99	0.99	1.00	1.00
	**p01**	**0.33**	**0.29**	**12**	**0.30**	**0.28**	**0.31**	**0.26**
$m = 4941$	*p02*	1.03	1.00	1	1.00	1.00	1.00	1.00
$n = 6594$	*p03*	0.44	0.29	12	0.44	0.30	0.25	0.15
	p04	0.55	0.38	16	0.55	0.48	0.15	0.33
	p05	0.45	0.34	35	0.34	0.40	0.45	0.16
	p06	1.02	0.99	1	1.00	0.99	1.00	0.99
	p07	1.02	1.00	1	1.00	0.99	1.00	1.00
	p08	0.59	0.44	16	0.56	0.58	0.32	0.30
	p09	1.18	1.10	7	1.12	1.12	0.98	1.18
	p10	1.02	1.00	1	1.00	1.00	1.00	1.00
	p11	1.02	1.00	1	1.00	0.99	1.00	1.00
	p12	1.02	1.00	1	1.00	1.00	1.00	0.99
	p13	0.34	0.27	13	0.30	0.31	0.32	0.15
	p14	1.02	1.00	1	1.00	1.00	1.00	0.99
	p15	0.84	0.62	13	0.44	0.41	0.78	0.84
	p16	1.02	0.99	1	0.99	0.99	1.00	0.99
	p17	1.02	0.99	1	0.99	0.99	1.00	1.00
	p18	0.54	0.38	8	0.42	0.38	0.54	0.20
	p19	1.01	0.99	1	0.99	0.98	1.00	0.99
	p20	1.02	0.99	1	0.99	1.00	1.00	0.98
	p21	0.49	0.34	16	0.28	0.49	0.23	0.38
	p22	1.36	1.13	1	1.10	1.10	0.97	1.36
	p23	1.02	0.99	1	0.99	0.99	1.00	1.00
	p24	1.02	0.99	1	0.99	0.99	1.00	0.99
	p25	1.02	1.00	1	1.00	0.99	1.00	0.99
	p26	1.02	0.99	1	0.99	0.99	1.00	1.00
	p27	1.02	1.00	1	0.99	1.00	1.00	0.99
	p28	1.02	1.00	1	1.00	0.99	1.00	1.00
	p29	1.02	1.00	1	1.00	1.00	1.00	1.00

**Proteins**<br><br>$m = 2967$<br>$n = 3576$	*p00*	0.72	0.57	16	0.35	0.69	0.54	0.70
	p01	0.74	0.59	18	0.46	0.74	0.57	0.58
	p02	0.89	0.61	11	0.29	0.82	0.85	0.47
	p03	0.92	0.60	17	0.33	0.34	0.92	0.80
	p04	0.74	0.45	19	0.24	0.53	0.31	0.74
	p05	1.02	1.00	1	1.01	1.01	1.00	1.00
	p06	0.64	0.44	11	0.25	0.51	0.63	0.38
	p07	1.01	1.00	1	1.00	1.00	1.00	1.00
	p08	1.01	1.00	1	1.00	1.00	1.00	1.00
	p09	0.70	0.58	10	0.63	0.68	0.35	0.67
	p10	0.53	0.43	10	0.46	0.45	0.52	0.30
	p11	1.02	1.00	1	1.00	1.00	1.00	1.00
	p12	0.49	0.38	15	0.42	0.36	0.48	0.25
	p13	0.87	0.73	10	0.56	0.86	0.85	0.65
	p14	0.86	0.56	12	0.49	0.72	0.17	0.86
	p15	0.49	0.39	11	0.47	0.47	0.22	0.39
	p16	0.48	0.36	16	0.35	0.48	0.31	0.31
	p17	0.80	0.52	16	0.29	0.37	0.80	0.63
	p18	0.60	0.41	14	0.60	0.41	0.23	0.41
	p19	0.85	0.77	11	0.71	0.72	0.84	0.79
	p20	0.43	0.30	29	0.19	0.35	0.24	0.42
	p21	0.97	0.72	19	0.70	0.44	0.82	0.92
	p22	0.63	0.51	17	0.51	0.52	0.54	0.48
	p23	0.92	0.64	7	0.44	0.35	0.89	0.91
	p24	0.53	0.33	7	0.22	0.53	0.19	0.38
	**p25**	**0.38**	**0.24**	**22**	**0.22**	**0.38**	**0.21**	**0.14**
	p26	0.90	0.62	4	0.34	0.46	0.79	0.89
	p27	0.44	0.30	24	0.31	0.44	0.17	0.26
	p28	0.68	0.49	8	0.45	0.66	0.67	0.17
	p29	1.03	0.99	1	0.95	1.00	0.99	1.03
**Word Co-occurrences**<br><br>$m = 112$<br>$n = 425$	*p00*	0.51	0.39	3	0.40	0.33	0.48	0.36
	p01	0.74	0.57	1	0.51	0.43	0.72	0.62
	p02	0.55	0.42	3	0.41	0.35	0.51	0.40
	p03	0.51	0.41	3	0.42	0.35	0.49	0.37
	p04	0.49	0.38	3	0.40	0.34	0.47	0.32
	p05	0.52	0.40	3	0.40	0.35	0.51	0.34
	p06	0.51	0.39	3	0.40	0.33	0.48	0.37
	p07	0.51	0.35	5	0.39	0.32	0.30	0.41
	p08	0.54	0.41	15	0.43	0.31	0.52	0.37
	p09	0.77	0.51	8	0.46	0.43	0.39	0.75
	p10	0.52	0.38	6	0.40	0.31	0.43	0.39
	p11	0.52	0.39	3	0.40	0.32	0.48	0.37
	p12	0.61	0.45	13	0.42	0.35	0.52	0.50
	p13	0.88	0.55	11	0.49	0.37	0.50	0.84
	p14	0.61	0.43	9	0.44	0.33	0.39	0.56
	p15	0.54	0.42	7	0.43	0.35	0.47	0.42
	**p16**	**0.48**	**0.38**	**3**	**0.39**	**0.33**	**0.45**	**0.35**
	p17	0.54	0.40	3	0.40	0.33	0.47	0.39
	p18	0.52	0.40	3	0.40	0.34	0.47	0.40
	p19	0.51	0.36	5	0.46	0.30	0.24	0.42
	p20	0.68	0.52	6	0.46	0.45	0.49	0.67
	p21	0.55	0.41	5	0.40	0.33	0.48	0.43
	p22	1.03	0.54	2	0.46	0.40	0.26	1.03
	p23	0.51	0.41	6	0.42	0.35	0.50	0.36
	p24	0.51	0.40	10	0.41	0.34	0.48	0.39
	p25	0.52	0.40	5	0.41	0.33	0.49	0.38
	p26	0.50	0.40	11	0.41	0.37	0.43	0.39
	p27	0.48	0.38	3	0.41	0.34	0.46	0.32
	p28	0.51	0.39	3	0.41	0.34	0.46	0.35
	p29	0.51	0.41	24	0.41	0.36	0.44	0.44

Table 5: Detailed relative results for undirected networks. Each line shows the mean values for 30 runs of one generator.

Network	Model				Distances						
	$prog$	f_{max}	f_{mean}	p_s	k_{in}	k_{out}	PR_d	PR_u	τ	d_d	d_u
**C. Elegans**	*p00*	0.53	0.32	25	515	346	797	617	18	0.80	0.51
	p01	0.56	0.42	14	551	256	1390	723	20	2.05	0.82
$m = 297$	*p02*	0.64	0.50	10	524	475	1482	953	25	1.99	1.05
$n = 2345$	*p03*	0.64	0.50	4	520	486	1506	942	25	2.07	1.05
	p04	0.51	0.38	13	376	280	1106	1181	19	0.87	1.03
	p05	0.75	0.57	9	745	612	1421	1341	26	2.33	0.77
	p06	0.46	0.28	16	275	331	724	491	17	1.12	0.44
	p07	0.82	0.54	3	418	695	819	611	32	2.78	1.30
	p08	0.62	0.49	10	505	472	1496	917	24	2.07	1.04
	p09	0.73	0.54	14	625	529	619	1011	28	2.35	1.50
	p10	0.64	0.41	8	654	318	1161	782	23	0.40	1.11
	p11	0.70	0.50	5	497	442	1132	1354	27	1.89	1.06
	p12	0.64	0.50	4	506	484	1505	942	25	2.10	1.05
	p13	0.87	0.55	5	464	600	826	772	34	2.86	1.27
	p14	0.59	0.46	14	492	433	1076	1396	22	1.83	0.82
	p15	0.82	0.50	9	571	277	1981	1029	32	1.80	0.77
	p16	0.61	0.41	45	458	269	1068	1024	24	1.52	0.87
	p17	0.59	0.47	23	547	484	902	974	22	1.92	1.16
	p18	0.81	0.54	5	416	650	929	732	32	2.67	1.30
	p19	0.61	0.47	21	599	472	1271	879	20	2.10	0.98
	p20	0.53	0.42	11	503	437	1048	878	17	1.78	1.01
	p21	0.68	0.46	11	663	454	949	940	26	1.85	0.75
	p22	0.83	0.53	23	541	454	1873	687	32	2.89	0.66
	p23	0.49	0.32	8	501	324	1067	898	15	0.39	0.59
	p24	0.89	0.51	13	678	607	515	679	29	1.66	1.48
	p25	0.46	0.28	13	293	286	656	514	17	1.71	0.26
	p26	0.54	0.43	9	562	319	1427	1136	14	2.01	0.87
	**p27**	**0.45**	**0.33**	**11**	**476**	**270**	**1101**	**828**	**12**	**1.31**	**0.52**
	p28	0.63	0.49	10	491	500	1362	815	24	2.28	1.10
	p29	0.79	0.61	7	762	603	2569	708	28	2.74	0.96
**Political Blogs**	*p00*	0.58	0.35	12	8076	7360	4863	5074	23	1.67	2.36
	p01	0.82	0.51	12	7653	10045	10350	16891	31	2.40	3.04
$m = 1490$	*p02*	0.59	0.33	28	6601	4547	2962	8783	24	2.17	2.37
$n = 19022$	*p03*	0.83	0.47	16	6175	9422	11517	9802	34	3.43	2.57
	p04	0.80	0.47	27	7787	9755	6636	11875	32	3.67	2.29
	p05	0.75	0.57	14	12373	12814	7871	15248	28	4.68	2.97
	p06	0.61	0.44	15	12738	6217	13224	9505	23	2.81	2.29
	p07	0.50	0.40	21	9228	5974	17798	9689	18	2.61	1.95
	p08	0.96	0.39	59	7429	4528	6875	8251	39	3.73	1.14
	p09	0.78	0.48	8	10269	4366	22606	13051	31	1.34	2.62
	p10	0.59	0.34	21	5976	7793	5899	5249	24	2.85	1.60
	**p11**	**0.42**	**0.27**	**18**	**5900**	**4892**	**9528**	**5456**	**17**	**1.02**	**1.63**
	p12	0.88	0.71	10	13229	13958	18010	18284	35	6.10	3.43
	p13	0.94	0.39	5	5191	2904	9966	11358	38	3.43	1.29
	p14	0.95	0.53	6	10241	12842	8987	13452	38	1.47	2.74
	p15	0.54	0.39	11	8460	4104	15261	12033	20	2.81	1.86
	p16	0.84	0.32	32	5593	4728	3121	7248	34	0.56	1.85
	p17	0.95	0.43	4	9176	7857	12898	10674	38	0.72	1.60
	p18	0.87	0.53	32	10824	9312	15373	10079	30	4.12	3.03
	p19	0.93	0.59	4	14536	13374	13580	12883	38	2.71	2.70
	p20	0.46	0.27	14	7165	6090	5395	4470	18	2.06	0.87
	p21	0.81	0.60	14	14239	10191	17275	13538	32	3.85	3.21
	p22	0.67	0.31	18	2302	7338	4257	8291	27	0.79	2.15
	p23	0.57	0.32	21	8319	7185	11233	4939	23	1.66	0.81

	p24	0.68	0.45	6	12292	6118	23705	13180	26	1.82	1.01
	p25	0.82	0.39	26	9583	3362	7882	7898	33	3.19	1.94
	p26	0.96	0.59	5	9811	12783	7803	14754	39	4.78	2.87
	p27	1.01	0.92	1	18288	17089	28083	25171	41	8.42	4.17
	p28	0.89	0.36	7	5713	6262	9074	8986	36	0.80	1.55
	p29	0.83	0.61	8	11560	13497	6165	18256	33	6.22	2.49
**Software Collaborations**	*p00*	0.96	0.66	8	1447	620	8321	5012	39	13.92	2.77
	p01	0.98	0.62	7	1907	596	7201	4477	39	15.26	1.50
	p02	0.80	0.47	22	1528	559	3596	4043	31	6.17	1.42
$m = 840$	*p03*	1.01	0.61	7	1873	595	6965	4492	40	12.88	1.57
$n = 2138$	*p04*	1.09	0.78	11	2356	657	10239	6574	43	21.32	2.21
	p05	0.92	0.51	9	1551	620	4616	4520	30	9.49	1.20
	p06	0.90	0.38	7	760	631	924	2248	34	1.87	1.22
	p07	0.97	0.70	3	2529	499	10688	5758	40	9.72	2.98
	p08	0.95	0.60	11	2171	665	5629	4777	38	7.50	1.96
	p09	0.91	0.50	11	1141	536	4835	3292	37	14.45	0.98
	p10	1.04	1.01	1	3301	723	17375	8912	42	23.50	3.95
	p11	1.01	0.80	12	3239	591	15332	7254	40	11.05	2.46
	p12	0.80	0.40	6	1386	555	2386	3166	29	4.97	0.73
	p13	0.93	0.54	5	2242	392	5731	4836	38	4.20	2.12
	**p14**	**0.69**	**0.33**	**12**	**1234**	**439**	**1107**	**2073**	**27**	**1.20**	**1.19**
	p15	0.96	0.76	15	2335	596	12720	6470	36	19.60	2.33
	p16	0.81	0.39	7	1324	442	1405	2575	33	1.58	1.66
	p17	0.85	0.37	6	1038	452	1791	2233	34	2.53	1.35
	p18	1.03	1.00	1	3298	715	17339	8840	40	24.12	3.85
	p19	0.89	0.55	5	1502	600	6416	5023	34	8.47	1.71
	p20	0.80	0.42	9	1856	495	2246	3351	31	4.17	0.92
	p21	0.94	0.45	16	651	615	3028	3844	35	5.75	1.32
	p22	1.03	1.00	1	3295	710	17324	8844	41	23.93	3.85
	p23	0.95	0.53	7	1500	665	5518	4344	34	9.78	0.99
	p24	0.80	0.34	19	1024	406	1044	2462	32	1.61	1.06
	p25	0.97	0.67	6	2110	649	10491	6471	39	6.23	2.24
	p26	1.01	0.95	1	3160	713	15230	8352	41	21.42	3.73
	p27	0.98	0.51	26	1726	524	4090	3198	39	12.93	0.85
	p28	1.03	1.00	1	3290	720	17325	8838	41	23.82	3.86
	p29	0.96	0.58	14	1271	442	7061	3554	39	18.85	1.97

Table 6: Detailed absolute results for directed networks. Each line shows the mean values for 30 runs of one generator.

**Network**	**Model**				**Distances**			
	$prog$	f_{max}	f_{mean}	p_s	k	PR	τ	d
**Facebook**	*p00*	0.64	0.56	13	22941	4857	2	2.86
	p01	0.62	0.48	7	18701	4720	2	1.99
$m = 747$	*p02*	0.77	0.56	9	22131	5837	2	1.58
$n = 30025$	*p03*	0.70	0.62	15	21476	4120	3	3.38
	p04	0.78	0.55	20	17366	2929	3	3.33
	p05	0.76	0.62	4	21163	5671	2	3.12
	p06	0.52	0.39	14	19458	2336	1	1.83
	p07	0.77	0.66	7	21540	4257	3	3.75
	p08	0.69	0.57	8	25522	4461	3	1.68
	p09	0.62	0.48	7	16526	4296	2	1.54
	p10	0.79	0.53	7	29456	4668	2	1.37
	p11	0.58	0.49	13	16253	3387	2	2.82
	p12	0.79	0.68	7	25932	4135	3	3.46
	**p13**	**0.29**	**0.20**	**14**	**8737**	**1060**	**1**	**0.87**
	p14	0.48	0.35	27	11280	3459	2	0.88
	p15	0.91	0.52	10	19005	6818	2	0.76

	p16	0.43	0.30	14	11671	2857	1	1.48
	p17	0.40	0.30	10	12447	2074	1	1.28
	p18	0.42	0.35	10	14427	2804	1	1.61
	p19	0.48	0.36	6	16779	3051	2	0.95
	p20	0.69	0.40	9	16448	5035	1	1.12
	p21	0.72	0.52	12	20812	5065	2	2.33
	p22	0.63	0.43	18	8134	2060	3	2.95
	p23	1.05	0.55	17	17028	7979	2	1.52
	p24	0.75	0.63	10	20277	5191	2	3.55
	p25	0.39	0.28	13	12164	2459	1	0.67
	p26	0.97	0.72	5	20109	4758	4	4.10
	p27	0.48	0.37	10	14046	3384	1	1.57
	p28	0.40	0.28	6	12788	1917	1	0.71
	p29	0.43	0.32	6	14236	2843	1	0.93
**Power Grid**	*p00*	1.02	1.00	1	1684	14530	3	5.06
	**p01**	**0.33**	**0.29**	**12**	**500**	**4090**	**1**	**1.32**
$m = 4941$	*p02*	1.03	1.00	1	1696	14635	3	5.07
$n = 6594$	*p03*	0.44	0.29	12	748	4447	1	0.77
	p04	0.55	0.38	16	936	7052	0	1.67
	p05	0.45	0.34	35	579	5910	1	0.79
	p06	1.02	0.99	1	1689	14465	3	5.00
	p07	1.02	1.00	1	1689	14529	3	5.05
	p08	0.59	0.44	16	943	8545	1	1.52
	p09	1.18	1.10	7	1896	16373	3	5.95
	p10	1.02	1.00	1	1686	14560	3	5.05
	p11	1.02	1.00	1	1695	14489	3	5.06
	p12	1.02	1.00	1	1700	14564	3	5.01
	p13	0.34	0.27	13	509	4486	1	0.77
	p14	1.02	1.00	1	1699	14558	3	5.03
	p15	0.84	0.62	13	752	6037	2	4.27
	p16	1.02	0.99	1	1682	14466	3	5.03
	p17	1.02	0.99	1	1681	14433	3	5.06
	p18	0.54	0.38	8	715	5484	2	0.99
	p19	1.01	0.99	1	1675	14396	3	5.03
	p20	1.02	0.99	1	1684	14556	3	4.98
	p21	0.49	0.34	16	466	7094	1	1.90
	p22	1.36	1.13	1	1859	16119	3	6.89
	p23	1.02	0.99	1	1679	14442	3	5.08
	p24	1.02	0.99	1	1677	14432	3	5.03
	p25	1.02	1.00	1	1690	14519	3	5.02
	p26	1.02	0.99	1	1683	14445	3	5.04
	p27	1.02	1.00	1	1682	14565	3	5.01
	p28	1.02	1.00	1	1685	14486	3	5.07
	p29	1.02	1.00	1	1686	14558	3	5.05
**Proteins**	*p00*	0.72	0.57	16	942	8679	2	1.40
	p01	0.74	0.59	18	1226	9310	2	1.16
$m = 2967$	*p02*	0.89	0.61	11	785	10329	4	0.93
$n = 3576$	*p03*	0.92	0.60	17	895	4222	4	1.58
	p04	0.74	0.45	19	645	6708	1	1.47
	p05	1.02	1.00	1	2690	12643	4	2.00
	p06	0.64	0.44	11	680	6400	3	0.76
	p07	1.01	1.00	1	2681	12581	4	1.99
	p08	1.01	1.00	1	2686	12555	4	1.98
	p09	0.70	0.58	10	1695	8497	1	1.34
	p10	0.53	0.43	10	1230	5643	2	0.59
	p11	1.02	1.00	1	2684	12592	4	1.99
	p12	0.49	0.38	15	1135	4529	2	0.49
	p13	0.87	0.73	10	1498	10764	4	1.30
	p14	0.86	0.56	12	1297	9083	1	1.71
	p15	0.49	0.39	11	1251	5849	1	0.78
	p16	0.48	0.36	16	938	6013	1	0.62

	p17	0.80	0.52	16	787	4625	3	1.25
	p18	0.60	0.41	14	1599	5091	1	0.81
	p19	0.85	0.77	11	1909	9039	3	1.57
	p20	0.43	0.30	29	508	4371	1	0.84
	p21	0.97	0.72	19	1864	5470	3	1.83
	p22	0.63	0.51	17	1360	6504	2	0.95
	p23	0.92	0.64	7	1172	4337	4	1.80
	p24	0.53	0.33	7	596	6701	1	0.75
	**p25**	**0.38**	**0.24**	**22**	**577**	**4735**	**1**	**0.29**
	p26	0.90	0.62	4	907	5760	3	1.78
	p27	0.44	0.30	24	830	5502	1	0.52
	p28	0.68	0.49	8	1194	8324	3	0.33
	p29	1.03	0.99	1	2540	12556	4	2.05
**Word Co-occurrences**	*p00*	0.51	0.39	3	115	351	1	0.17
	p01	0.74	0.57	1	147	461	2	0.29
	p02	0.55	0.42	3	120	377	2	0.18
$m = 112$	*p03*	0.51	0.41	3	121	372	1	0.17
$n = 425$	*p04*	0.49	0.38	3	117	368	1	0.15
	p05	0.52	0.40	3	117	373	2	0.16
	p06	0.51	0.39	3	116	356	1	0.17
	p07	0.51	0.35	5	112	346	1	0.19
	p08	0.54	0.41	15	124	329	2	0.17
	p09	0.77	0.51	8	133	461	1	0.34
	p10	0.52	0.38	6	115	330	1	0.18
	p11	0.52	0.39	3	115	340	1	0.17
	p12	0.61	0.45	13	121	375	2	0.23
	p13	0.88	0.55	11	143	393	2	0.39
	p14	0.61	0.43	9	129	351	1	0.26
	p15	0.54	0.42	7	125	374	1	0.19
	**p16**	**0.48**	**0.38**	**3**	**113**	**349**	**1**	**0.16**
	p17	0.54	0.40	3	115	351	1	0.18
	p18	0.52	0.40	3	116	362	1	0.18
	p19	0.51	0.36	5	133	317	1	0.19
	p20	0.68	0.52	6	135	485	1	0.31
	p21	0.55	0.41	5	117	349	1	0.20
	p22	1.03	0.54	2	135	429	1	0.47
	p23	0.51	0.41	6	120	370	2	0.17
	p24	0.51	0.40	10	118	364	1	0.18
	p25	0.52	0.40	5	118	351	1	0.17
	p26	0.50	0.40	11	120	391	1	0.18
	p27	0.48	0.38	3	119	358	1	0.15
	p28	0.51	0.39	3	119	360	1	0.16
	p29	0.51	0.41	24	119	390	1	0.20

Table 7: Detailed absolute results for undirected networks. Each line shows the mean values for 30 runs of one generator.

References and Notes for Supplementary Information

1. Ling, H., Okada, K. An efficient Earth Mover's Distance algorithm for robust histogram comparison. *IEEE Trans. Pattern Anal. Mach. Intell.* **29**, 840–853 (2007).

2. Koza, J. R. *Genetic programming: on the programming of computers by means of natural selection* (MIT Press, Cambridge, MA, USA, 1992).

3. Poli, R., Langdon, W. B., McPhee, N. F. *A Field Guide to Genetic Programming.* (Lulu.com, 2008). Date of access:10/03/2014

4. Bailey, A., Ventresca, M., Ombuki-Berman, B., [Automatic generation of graph models for complex networks by genetic programming] *Proc. 14th international conference on Genetic and evolutionary computation conference (GECCO '12)* [711–718] (ACM, New York, 2012).

5. Bailey, A., Ventresca, M., Ombuki-Berman, B., Genetic Programming for the Automatic Inference of Graph Models for Complex Networks, *IEEE Tran. Evo. Comp.* **18**, 405–419 (2013).

6. Kossinets, G., Watts, D. J., Empirical Analysis of an Evolving Social Network, *Science*, **311**, 88–90 (2006).
